# PARVA Promotes Metastasis by Modulating ILK Signalling Pathway in Lung Adenocarcinoma

**DOI:** 10.1371/journal.pone.0118530

**Published:** 2015-03-04

**Authors:** Ay-Huey Huang, Szu-Hua Pan, Wen-Hsin Chang, Qi-Sheng Hong, Jeremy J. W. Chen, Sung-Liang Yu

**Affiliations:** 1 Institute of Molecular Biology, National Chung Hsing University, Taichung, Taiwan; 2 Graduate Institute of Medical Genomics and Proteomics, National Taiwan University College of Medicine, Taipei, Taiwan; 3 Institute of Biomedical Sciences, National Chung Hsing University, Taichung, Taiwan; 4 Agricultural Biotechnology Center, National Chung Hsing University, Taichung, Taiwan; 5 Department of Clinical Laboratory Sciences and Medical Biotechnology, College of Medicine, National Taiwan University, Taipei, Taiwan; 6 Center of Genomic Medicine, National Taiwan University, Taipei, Taiwan; 7 Center for Optoelectronic Biomedicine, College of Medicine, National Taiwan University, Taipei, Taiwan; 8 Department of Laboratory Medicine, National Taiwan University Hospital, Taipei, Taiwan; West Virginia University, UNITED STATES

## Abstract

α-parvin (PARVA) is known to be involved in the linkage of integrins, regulation of actin cytoskeleton dynamics and cell survival. However, the role that PARVA plays in cancer progression remains unclear. Here, using a lung cancer invasion cell line model and expression microarrays, we identify PARVA as a potential oncogene. The overexpression of PARVA increased cell invasion, colony-forming ability and endothelial cell tube formation. By contrast, knockdown of PARVA inhibited invasion and tube formation *in vitro*. Overexpression of PARVA also promoted tumorigenicity, angiogenesis and metastasis in *in vivo* mouse models. To explore the underlying mechanism, we compared the expression microarray profiles of PARVA-overexpressing cells with those of control cells to identify the PARVA-regulated signalling pathways. Pathway analysis showed that eight of the top 10 pathways are involved in invasion, angiogenesis and cell death. Next, to identify the direct downstream signalling pathway of PARVA, 371 significantly PARVA-altered genes were analysed further using a transcription factor target model. Seven of the top 10 PARVA-altered transcription factors shared a common upstream mediator, ILK. Lastly, we found that PARVA forms a complex with SGK1 and ILK to enhance the phosphorylation of ILK, which led to the phosphorylation of Akt and GSK3β. Notably, the inactivation of ILK reversed PARVA-induced invasion. Taken together, our findings imply that PARVA acts as an oncogene by activating ILK, and that this activation is followed by the activation of Akt and inhibition of GSK3β. To our knowledge, this is the first study to characterize the role of PARVA in lung cancer progression.

## Introduction

Lung cancer is the leading cause of cancer-related death worldwide [1]. Metastasis and tumorigenesis are two major determinants that contribute to cancer progression and cancer-related death [2,3]. Invasion is the first crucial step of metastasis. Metastasis is a complex process involving malignant transformation that facilitates metastasis to distal organs [3,4]. Many important components of cell–matrix adhesion contribute to cell survival, growth, metastasis and carcinogenesis [5,6,7,8]. Because the signalling pathways that respond to stimulation of the extracellular matrix (ECM) become dysregulated in malignant cells, investigation of ECM-related genes may lead to a better understanding of the molecular regulation of tumorigenesis and metastasis, and may improve the development of targeted molecular therapies.

Upon integrin engagement with the ECM, α-parvin (PARVA) and PARVA-interacting proteins, including integrin-linked kinase (ILK) and particularly interesting new Cys-His protein (PINCH), preassemble into PARVA–ILK–PINCH (PIP)-containing multiprotein complexes that are recruited to nascent ECM–cell contacts, a process that is followed by interaction with paxillin, integrins and/or Mig-2 [9]. PIP complexes are essential for the establishment and maturation of focal adhesions, which provide the physical link between activated integrins and the actin cytoskeleton and which mediate signal transduction from the ECM via intracellular signalling pathways that regulate cell morphology, motility and survival. ILK has been characterized as an oncogene that exhibits protein kinase activity, results in the activation of PKB/Akt and GSK3β and finally contributes to PI3K-mediated integrin engagement, cell invasion and tumorigenicity [10,11,12].

The formation and composition of PIP complexes represent key determinants that govern which downstream signalling pathways are activated and which cellular functions are consequently altered. Disruption of PIP complexes by TGF-β 1 has been shown to activate the mitogen-activated protein kinase (MAPK) p38 and to promote apoptosis in podocytes. LNK (Src homology 2-B3; SH2B3) has been identified as an ILK-binding partner that interacts with ILK, and this interaction results in the downregulation of PARVA expression and a decrease in the adhesion and migration of endothelial cells [13]. PARVA is an important component of cell–matrix adhesions, which are involved in the proliferation, spreading and migration of human cells [11,14,15,16,17]. However, the role of PARVA in cancer progression has not been explored thoroughly. A recent report showed that PARVA expression correlated positively with ILK expression in chondrosarcomas [6]. Another study reported that PARVA phosphorylation was necessary for matrix degradation and for promotion of cell invasion via the activation of Src and matrix metalloproteinase (MMP) in osteosarcoma and breast cancer cells [18].

In this study, to focus on the discovery of cancer-related genes, we compared the RNA expression profiles of lung cancer cell lines exhibiting high and low rates of invasion. We used *in vitro* and *in vivo* metastasis and tumorigenesis assays to identify *PARVA* as an oncogene in lung adenocarcinoma. The mechanism underlying the role of PARVA in lung cancer progression was revealed by expression microarrays and pathway analysis. To our knowledge, our study is the first to characterize the oncogenic role of PARVA in lung cancer.

## Materials and Methods

### Cell lines and culture conditions

The human lung adenocarcinoma CL1–0 and CL1–5 cell lines, which exhibit varying invasive capacities, were established and characterized as described previously [19]. The human lung adenocarcinoma A549 cell line was purchased from the American Type Culture Collection (ATCC CCL-185) and was subjected to STR-PCR profiling at the Bioresource Collection and Research Center (Shinchu, Taiwan). All of the cell lines were cultured in RPMI-1640 medium (Life Technologies, Carlsbad, CA) supplemented with 10% foetal bovine serum (FBS), 100 U/ml penicillin and 100 μg/ml streptomycin (Life Technologies) at 37°C in a humidified atmosphere containing 5% CO_2_.

### Expression vector construction and transfection

First-strand CL1–5 cDNAs were reverse-transcribed using Blend Taq reverse transcriptase (Life Technologies) and Random Hexamer primers (Life Technologies). The PARVA-coding region (GenBank NM_018222) was amplified by polymerase chain reaction (PCR) using the 5′-CTGCGCCGCGCCATGGCCACCTC-3′ (forward) and 5′-CTCCACGTTACGGTACTTGGTGAAG-3′ (reverse) primers. The PCR products were cloned into the constitutive mammalian expression vector pcDNA3.1/V5-His-TOPO, whose expression is under the control of a CMV promoter (Life Technologies). The resulting vector, pcDNA3.1-PARVA, was sequence verified. Subsequently, the CL1–0, CL1–5 and A549 cells were transfected with either pcDNA3.1-PARVA or pcDNA3.1 vector using a Lipofectamine 2000 Transfection Reagent or LTX kit (Life Technologies), according to the manufacturer’s protocol. The pooled, stable PARVA-overexpressing cells were selected using 1 mg/ml G418 (Geneticin; Life Technologies) for the CL1–0 and A549 cells or 2 mg/ml G418 for the CL1–5 cells.

### Lentiviral shRNA–mediated knockdown

Luciferase (pLKO.1-shLuc; TRC001) and 2 PAK1-shRNA–containing lentiviral vectors (pLKO.1 shPAK1–1 and pLKO.1-shPAK1–2; TRCN0000002226 and TRCN0000002227) were obtained from the National RNAi Core Facility (Academia Sinica, Taipei, Taiwan) and prepared in accordance with the standard protocols. Cells were infected with lentivirus (multiplicity of infection 5) in medium containing polybrene (8 mg/ml). At 24 hours after infection, cells were treated with 2.5 mg/ml puromycin to select for puromycin-resistant pooled clones for further assays.

### Western blotting

Cells were lysed in lysis buffer (0.1% NP-40, 1× PBS), and the protein concentration of the resulting supernatants was measured using a BCA protein assay kit (Thermo Scientific, Rockford, IL). Forty micrograms of proteins were resolved in 1× protein sample buffer and separated on 8–12% SDS–polyacrylamide gels, and the bands were transferred to a PVDF membrane (Amersham BioSciences, Little Chalfont, Bucks, UK). After blocking in TBST (0.1% Tween-20 in TBS) supplemented with 5% skim milk, the membrane-bound proteins were probed with the indicated specific primary and secondary antibodies, visualized using ECL Western blot detection reagents (Millipore, Darmstadt, Germany) and detected by the LAS-3000 ECL system (Fujifilm, Tokyo, Japan). The primary antibodies used for Western blot analysis were V5 (Life Technologies), α-parvin (Cell Signaling Technology, Frankfurt am Main, Germany), ILK (Cell Signaling), phospho-ILK (Ser246, Abcam, Cambridge, MA; or T173, Cell Signaling), Akt and pAkt (Cell Signaling), GSK3β and pGSK3β (Ser9) (Millipore), p-c-Jun (Ser63/73), VEGF-A (Santa Cruz Biotechnology, Santa Cruz, CA), c-Jun (GeneTex, San Antonio, TX), SGK1 (GeneTex), α-tubulin and β-actin (Sigma-Aldrich, St. Louis, MO). The Western blot experiments were repeated at least three times. Where appropriate, the Western blot data were quantified by a publicly available software (Image J; NIH, Bethesda, MD) and normalized with the loading control.

### Co-immunoprecipitation

Cell lysates were prepared with immunoprecipitation (IP) lysis buffer (20 mM Tris pH 7.5, 100 mM NaCl, 1% Nonidet P40, 100 μM Na_3_VO_4_, 50 mM NaF, 30 mM sodium pyrophosphate) supplemented with 1× complete protease inhibitor cocktail (EDTA-free) (Roche Diagnostics GmbH, Penzberg, Germany), followed by thorough shearing using a 21-gauge needle. The samples were centrifuged, and the resulting protein lysate (1 mg) was incubated overnight at 4°C with either 2 μg of anti-V5 antibody (Life Technologies) or 2 μg of anti-ILK antibody (Cell Signaling). The samples were incubated for 2 h at 4°C with protein A/G agarose beads (Santa Cruz), and the bound beads were washed three times with IP lysis buffer. The protein samples were supplemented to 1× with protein sample buffer, boiled, resolved by 8% SDS–PAGE and transferred to a PVDF membrane (Amersham). The membrane-bound proteins were then subjected to Western blot analysis.

### Quantitative real-time RT-PCR

The expression of PARVA was detected by SYBR Green real-time RT-PCR using an ABI prism 7900 sequence detection system. TATA-box binding protein (GenBank NM_003194; TBP) was used as an internal control. The primers were as follows: PARVA forward primer 5′-ACGGTACCATGGCCCCTCCC-3′, PARVA reverse primer 5′-AGTCTAGACTCCACGTTACGGTA-3′, TBP forward primer 5′-CACGAACCACGGCACTGATT-3′ and TBP reverse primer 5′-TTTTCTTGCTGCCAGTCTGGAC-3′. The SYBR Green PCR Master Mix reagent (Life Technologies) was used to measure the increase in fluorescence throughout the PCR cycles. The relative expression levels of PARVA were determined as—ΔCT = –[CT_PARVA_–CT_TBP_]. The PARVA/TBP mRNA ratio was calculated as 2^–ΔCT^×K, where K is a constant.

### Migration and invasion assays

The migration and invasion activities of the transfectants were measured using Boyden chamber assays with Transwell plates (8-μm pore size; Corning Costar, Cambridge, MA). For the *in vitro* cell migration assay, 1×10^5^ cells were seeded into the Transwell inserts in 200 μl of serum-free medium. The lower wells of the Transwell inserts contained medium supplemented with 10% FBS. After 8 h of incubation, the inserts were fixed with methanol, swabbed and stained with Giemsa solution (Sigma-Aldrich). The number of migrated cells was counted using a light microscope at 200× magnification. The experiments were performed in triplicate independently. For the *in vitro* invasion assay, the inserts were coated with Matrigel (2.5 mg/ml; BD Biosciences, Bedford, MA). Next, 1×10^5^ cells (CL1–0) or 1×10^4^ cells (CL1–5 and A549) were seeded onto the Transwell inserts for 18 h. The subsequent steps were identical to those of the migration assay described above.

### Colony-forming assay

In the anchorage-independent growth assay, the bottom layer of the six-well plates contained 0.7% agarose in PBS, and the top layer contained 0.3% agarose in medium supplemented with 10% FBS. Cells were suspended in 1 ml of RPMI containing 0.35% low-melting-point agarose and were seeded onto the top layer of agarose at a density of 500 cells/well. After 2 weeks, the wells were washed in PBS, fixed in 4% paraformaldehyde and stained with 0.1% crystal violet. Colonies with a diameter >0.5 mm in the six wells were counted in two independent experiments. In the anchorage-dependent growth assay, cells were seeded at a density of 80 cells in 2 ml of culture medium in each of the wells of a six-well plate. Ten days later, the colonies were fixed, stained and counted.

### *In vivo* mouse model

Six-week-old non-obese diabetic–severe combined immunodeficiency (NOD/SCID) mice were supplied by the National Laboratory Animal Center, Taipei, Taiwan, and housed in specific pathogen-free animal rooms. All animal experiments were approved by the National Taiwan University College of Medicine and College of Public Health Institutional Animal Care and Use Committee (Permit Number: 20080214). For the orthotopic tumour implantation assay, 1×10^5^ cells in 20 μl of PBS containing 10 ng of Matrigel were implanted into the pleural cavity of each adult NOD/SCID mouse. After 4 weeks, the mice were sacrificed with carbon dioxide. The lungs and other organs were removed, fixed in 10% formalin and stained with Bouin solution [20]. The number of tumour colonies in the lung was counted using a dissecting microscope. For the tumorigenesis assay, 1×10^6^ cells were suspended in 100 μl of PBS and injected subcutaneously into each mouse. The tumour volume was measured every 3–5 days. After 4 weeks, the mice were sacrificed, and the tumours were harvested and weighed. The embedded tissues were sliced into 4-μm thick sections and stained with hematoxylin–eosin for histological analysis.

### Evaluation of microvessel density (MVD) and tube formation

To evaluate the MVD in the lung tumours, CD31 antibody was used to identify the microvessels. The detailed procedures and calculations have been described previously [21]. The vessel visualization index (VVI) was used to score MVD and was determined by assessing each entire core at 200× magnification. CD31^+^ angiogenesis was categorized as ‘high’, ‘moderate’ or ‘low’ by two blinded observers to obtain a consensus categorization. Clusters of stained endothelial cells that were distinct from adjacent microvessels, tumour cells or other stromal cells were counted as one microvessel. For statistical analysis, a combined score was created from the CD31^+^ staining and VVI as follows: 1 = high vessel tumours, and 0 = moderate or low vessel tumours. The MVD of tumours derived from mock or PARVA transfetant was calculated as the mean of three individual immunohistochemistry slides from three different mice, respectively. Each slide represents the average value of microvessel numbers of five fields. For the tube formation assay, human umbilical vein endothelial cells (passage 3, Part# C2519A, Lonza, Walkersville MD) were used in the experiment. First, 20 μl of Matrigel was added to each well of 96-well plates for 30 min. Five hundred cells were seeded per well, and the plate was incubated for 4–6 h in serum-free conditioned media collected from different transfectants including mock and stably pooled PARVA-overexpressing cells. Cells were photographed at low power (100×). The branch points and the total tube lengths (μm) were measured using the MetaXpress program (Molecular Devices, Sunnyvale, CA).

### Zymography assay

One million cells were seeded into six-well plates and incubated for 12 h at 37°C to 80% confluence. The cells were washed twice with PBS buffer and cultured for an additional 24 h in serum-free medium, and the resulting supernatants were collected. The activities of MMP2 and MMP9 in the supernatant were analysed by 8% SDS–PAGE with 0.5 mg/ml gelatine. The gels were washed twice for 30 min at room temperature in washing buffer, incubated in activation buffer for 24 h at 37°C and stained with 0.1% Coomassie brilliant blue R-250.

### Microarray and pathway analysis

The cRNA preparation and array hybridization were performed according to the protocols of the Affymetrix GeneChip Expression Analysis Technical Manual. All of the experiments were performed in triplicate using cRNA probes prepared from mock or constitutively PARVA-overexpressing CL1–0 cells. The microarray data were filtered by 1.5-fold changes under FDR protection (*P* < 0.05) by JMP Genomics software (SAS Inc., Cary, NC). The detailed procedures have been described previously [22]. Genes whose expression differed between the groups were subjected to pathway analysis using MetaCore software (GeneGo, St. Joseph, MI).

### Statistical analysis

All of the *in vitro* experiments were performed in three sets of triplicates, with the exception of the colony-forming assays. Significant differences were identified by *t* test (Excel, Microsoft, New York, NY). *P* < 0.05 was considered significant. Where appropriate, the results are presented as the mean values ± SD.

## Results

### PARVA promotes the migration and invasion of lung cancer cells

**Fig 1:**
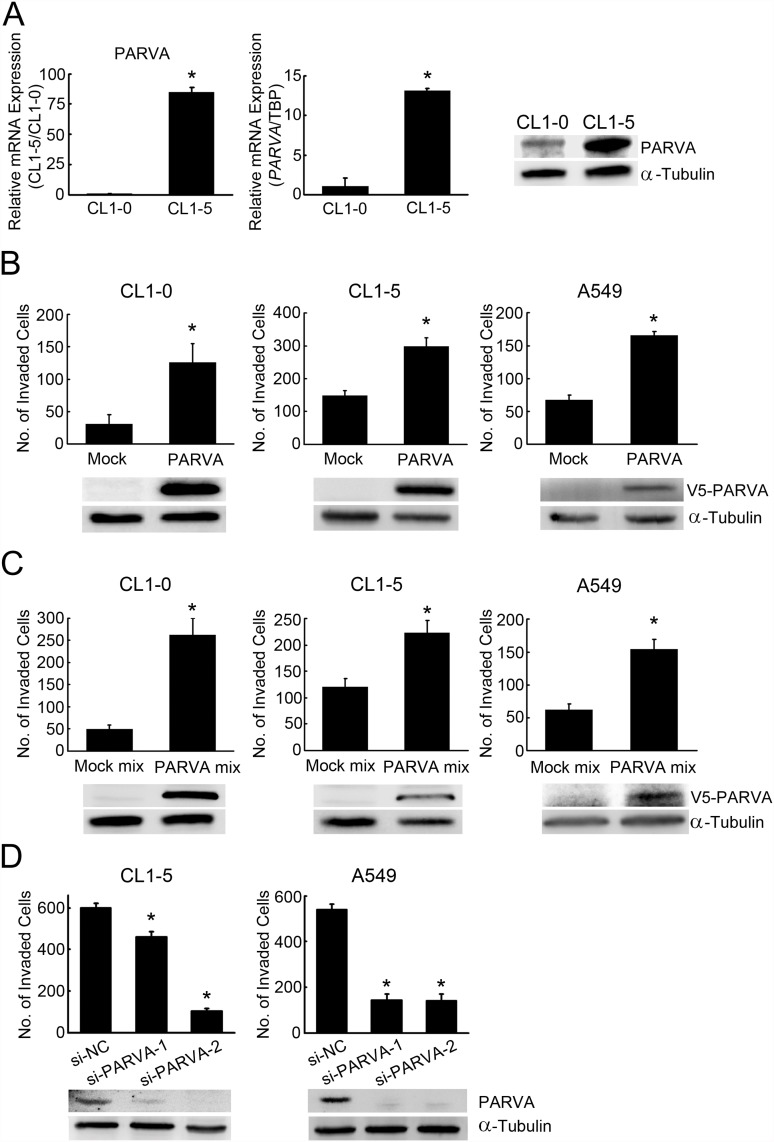
PARVA increases invasiveness in lung cancer cells. (A) PARVA is upregulated in highly invasive CL1–5 lung cancer cells compared with the less invasive CL1–0 cells. PARVA RNA and protein expression levels as determined by expression microarrays (left panel), real-time RT-PCR (middle panel) and Western blotting (right panel). (B) The effects of transient PARVA overexpression on cell invasion. CL1–0, CL1–5 and A549 cells were transiently transfected with the pcDNA3.1/V5-His TOPO-tagged PARVA construct or an empty vector, and the invading cells were evaluated by Boyden chamber assays. (C) The *in vitro* invasion ability of pooled, stably PARVA-overexpressing cells. CL1–0, CL1–5 and A549 cells were stably transfected with the pcDNA3.1/V5-His TOPO-tagged PARVA construct (PARVA mix) or an empty vector (Mock mix), and their invasive abilities were determined by Boyden chamber assays. (D) The *in vitro* invasion assay of PARVA-knockdown cells. Endogenous levels of PARVA were transiently silenced by two siRNAs, si-PARVA-1 and si-PARVA-2, in CL1–5 and A549 cells, and were detected using anti-PARVA antibodies. The invasive ability of the cells was analysed by Boyden chamber assays at 48 h after transfection. *, *P* < 0.05, compared with the mock control cells. The invasion data represent the average of three individual well counts ± SD. α-Tubulin was used as the loading control.

In our previous studies, we used expression microarrays to identify many potential metastasis-related genes from a lung cancer cell line model that exhibited varying invasion capacities. Among these genes, PARVA was identified as an ideal candidate for further investigation of its role in lung cancer progression because of the significant differences in expression between the highly invasive CL1–5 and less invasive CL1–0 cells as determined by microarray analysis (Fig. 1A, left panel) [23,24]. The differential expression of PARVA was verified by real-time RT-PCR and Western blot analysis (Fig. 1A, middle and right panels). To explore the cellular functions of PARVA in lung cancer progression, three constitutively PARVA-overexpressing cell lines, CL1–0, CL1–5 and A549, were generated to minimize the potential bias derived from the different genetic backgrounds of cell lines. The overexpression of PARVA was verified by real-time RT-PCR (S1A Fig.). The migration assays showed that PARVA significantly promoted the ability of lung cancer cells to migrate compared with the mock control cells (S1B Fig.).

We evaluated the contribution of PARVA to cell invasion. Similarly to the Boyden Chamber migration assay results, PARVA significantly increased the invasive ability of all lung cancer cell lines examined, CL1–0, CL1–5 and A549, by 2–3-fold compared with the mock control cells. This effect was observed regardless of whether the PARVA overexpression was transient (Fig. 1B) or stable (Fig. 1C). To determine whether PARVA-mediated migration and invasion could be attributed at least partly to increased cell proliferation, we performed a cell proliferation assay. PARVA did not affect the proliferation of CL1–5 and A549 cells (S2 Fig.). Next, we performed RNA silencing experiments to evaluate the specificity of PARVA for cell invasion. To reduce the potential off-target effect of the siRNAs, two PARVA-specific siRNAs were used to knock down PARVA expression in two highly PARVA-expressing cell lines. The reduction in PARVA expression decreased the rate of invasion (Fig. 1D).

### PARVA increases tumour cell growth *in vitro and in vivo*

**Fig 2:**
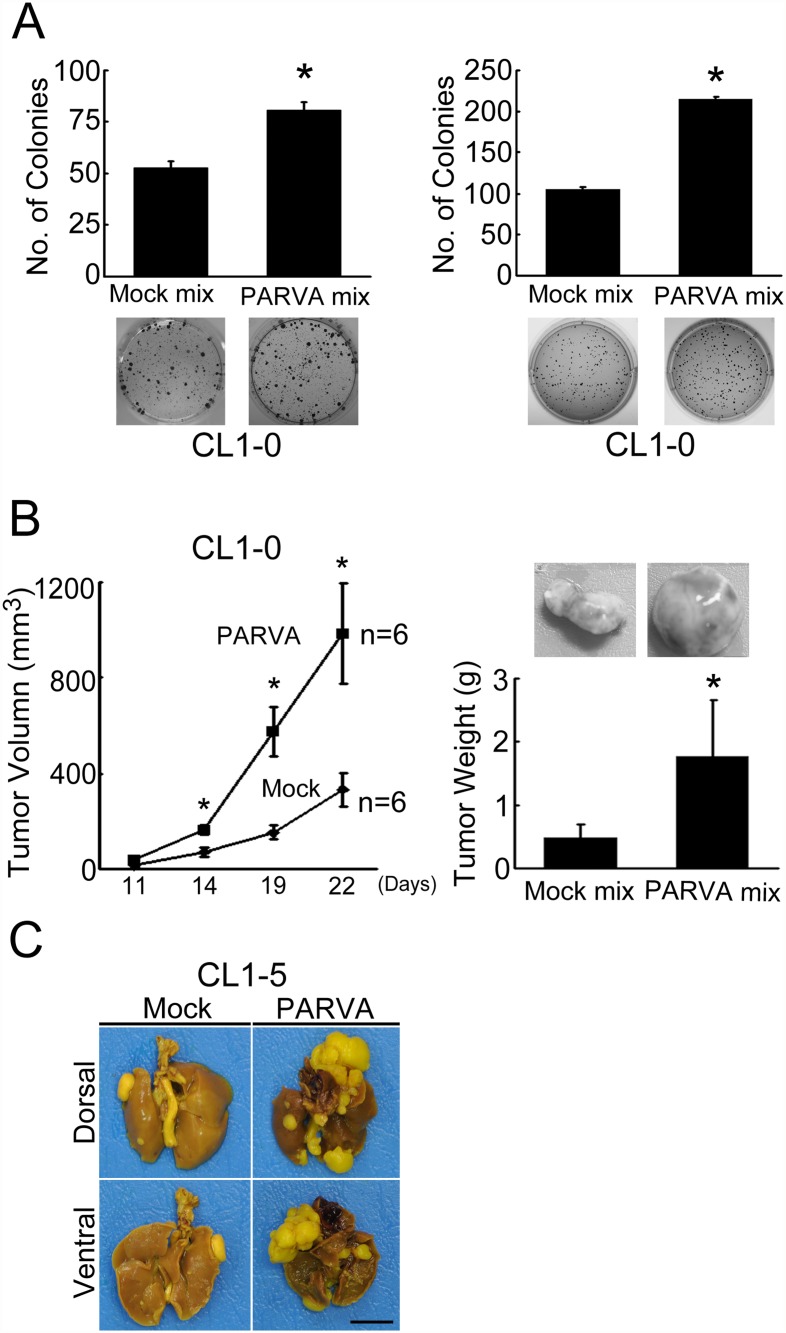
PARVA increases tumorigenicity and metastasis in lung cancer *in vitro* and *in vivo*. (A) PARVA increases clonogenesis *in vitro*, as measured by colony-forming assays. Both the mixed populations of mock-transfected cells (mock mix) and PARVA-transfected cells (PARVA mix) were derived from CL1–0 cells. Eighty cells were seeded for the anchorage-dependent assays (left panel) and 500 cells were seeded for the anchorage-independent assays (right panel). Colonies with a diameter >0.5 mm were counted using an inverted microscope. Each value of bar is presented as the average of six wells. *, *P* < 0.05, compared with the mock control. (B) PARVA increases tumorigenesis *in vivo*. One million mock-transfected (mock mix) and PARVA-overexpressing (PARVA mix) cells were injected subcutaneously into NOD/SCID mice. The tumour volume was measured every 3 or 4 days and was calculated using the following formula: *V = A* (length) × *B* ^2^/2 (width) (left panel). Tumour weight was measured in the excised tumours (right panel). Tumour volume and weight are presented as the average values of six individual mice ± SEM. A representative image of tumours excised from mock and pooled PARVA-overexpressing mice is shown in the right panel. Scale bar: 5 mm. *, *P* < 0.05, compared with the mock control group. (C) A representative example of lungs in the mouse metastasis model. Mock mix and PARVA mix stable clones were implanted (orthotopic injection) into the left lobe of the lung of NOD/SCID mice. After 4 weeks, the mice were sacrificed, and the lungs were stained with Bouin solution and photographed. Scale bar: 10 mm.

Both *in vitro* and *in vivo* approaches were taken to investigate whether PARVA is involved in tumorigenesis. The anchorage-dependent colony-forming assay showed that the ability of the pooled PARVA-overexpressing stable transfectants derived from CL1–0 cells to colonize in a 2D culture environment was significantly increased compared with the mixed population of mock-transfected cells (Fig. 2A, left panel). Similar results were obtained in the anchorage-independent assay in which the cancer cells were stretched fully within the 3D environment of the soft agar (Fig. 2A, right panel).

To confirm the tumorigenic activity of PARVA *in vivo*, the mixed population of PARVA-transfected CL1–0 cells was injected subcutaneously into NOD/SCID mice. Compared with the mock control cells, the PARVA-overexpressing tumour volume was significantly larger at day 14 and later (Fig. 2B, left panel). The weight was also greater in the PARVA-overexpressing tumours compared with the mock tumours (Fig. 2B, right panel). These results indicated that PARVA increased tumour growth *in vivo*.

### PARVA increases lung cancer metastasis *in vivo*

To assess whether PARVA is involved in the regulation of lung cancer metastasis, a mouse metastasis model with orthotopic implantation was performed. The CL1–5 mock and PARVA transfectants were implanted into the left lung lobes in NOD/SCID mice. After 4 weeks, the mice were sacrificed. Nodules formed on the left lobe, original inoculation site and the other four lobes of the right lung were counted separately. The mean ± SD numbers of nodules were 7.5 ± 4.0 and 4.8 ± 1.9 in the mock and PARVA mice, respectively. PARVA overexpression induced nodule formation in the right lung lobes (Fig. 2C) and resulted in metastasis to other distal organs in the thoracic cavity, including the trachea, pleura, heart and diaphragm (Table 1). The number of tumours formed on one of the other thoracic organs was counted. PARVA transfectant-implanted mice exhibited more incidences of distant metastasis compared with the control mice (4/6 vs. 2/6). The mice transplanted with the PARVA-overexpressing cells died earlier than the mice transplanted with the mock cells. The 4-week mortality rate was greater in the PARVA-overexpressing group compared with the control group (3/6 vs. 0/6). These data implied that PARVA increased metastasis *in vivo* and shortened mouse survival.

**Table 1:** A comparison of the metastatic incidence between mock and PARVA transfectants in an orthotopic lung implantation mouse model.

Cell	No. of metastatic nodules at right lung		Incidence of distant metastasis^#^	Mortality rate
	Mean ± SD	*P*	Other organs*	
CL1–5/Mock (n = 6)	4.8±1.9		2/6	0/6
CL1–5/PARVA (n = 6)	7.5±4.0	< 0.05	4/6	3/6

*Other organs: trachea, pleura, heart and diaphragm

^#^Incidence: affected mice/ total mice.

### PARVA is involved in the regulation of angiogenesis

**Fig 3:**
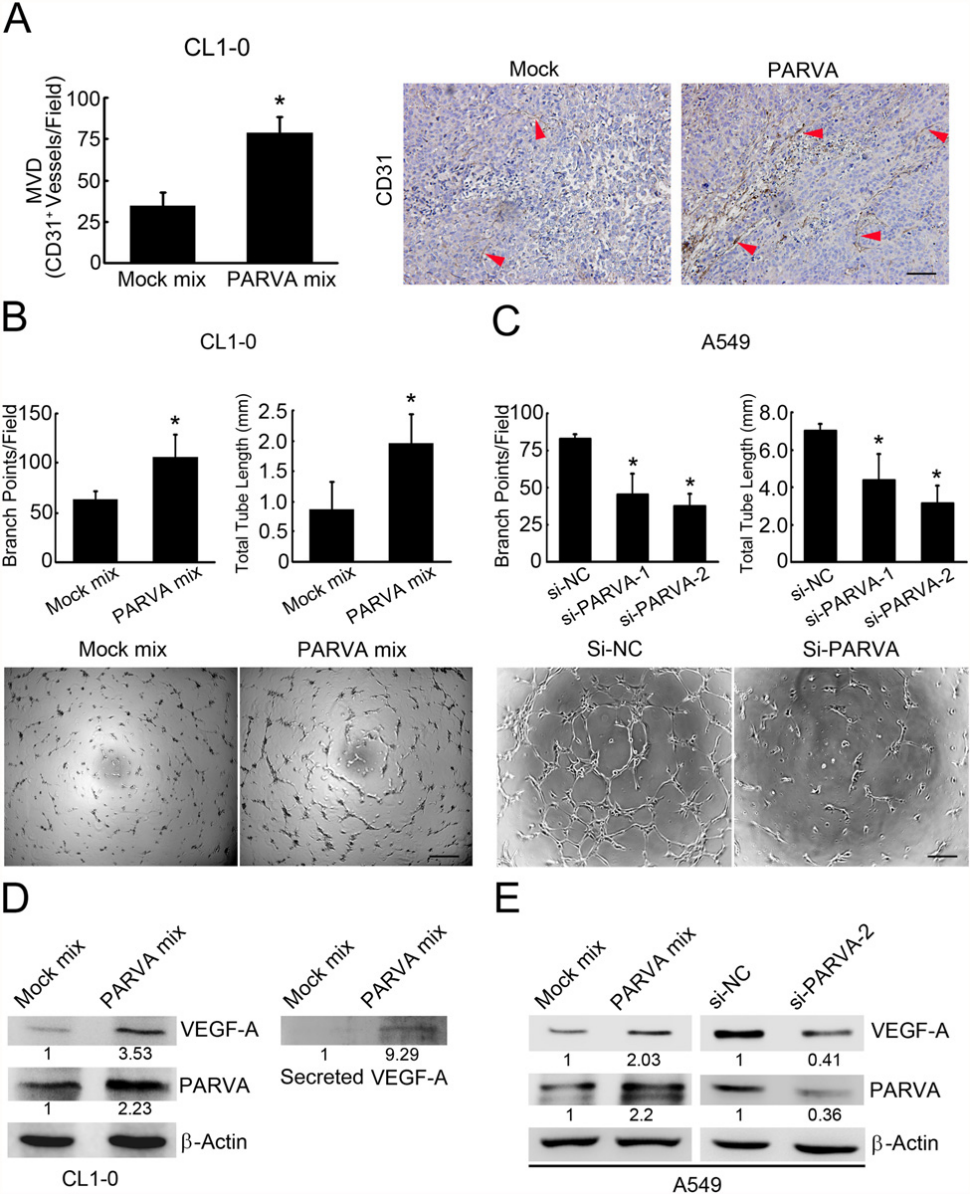
PARVA promotes tube formation *in vitro* and angiogenesis *in vivo*. (A) The effects of PARVA on angiogenesis *in vivo*. Mock and PARVA-overexpressing transfectants derived from CL1–0 cells were inoculated into NOD/SCID mice as mentioned in Fig. 2B. The resulting tumours were sectioned and stained with an anti-CD31 antibody. A representative immunohistochemistry image is shown in the right panel. The microvessel density (MVD) for each group represents the mean value of three individual immunohistochemistry slides from three different mice. Each slide represents the average value of microvessel numbers of five fields (left panel). The arrowheads indicate CD31^+^ vessels. Scale bar: 10 μm. *, *P* < 0.05, compared with the mock control groups. (B) Induction of tube formation by PARVA. Endothelial cells were treated for 4 h with serum-free culture medium from mock or stably pooled PARVA-overexpressing CL1–0 transfectants, and the branch points and tube lengths were counted using the angiogenesis module of MetaXpress. Each experiment was performed in triplicate. A representative image of the tube formation assay is shown in the lower panel. Scale bar: 10 μm. *, *P* < 0.05, compared with the mock control groups. (C) Reduction in tube formation induced by PARVA silencing. Endothelial cells were treated with the serum-free culture medium from the scrambled control (si-NC) and PARVA-silenced A549 transfectants (si-PARVA-1 and si-PARVA-2), and the rate of tube formation was determined as described in (C). *, *P* < 0.05, compared with the scrambled control (si-NC). (D) Induction of VEGF-A by PARVA. The cellular and secreted forms of VEGF-A were detected in the mock and stably PARVA-overexpressing CL1–0 transfectants by Western blotting. β-Actin was used as the loading control. (E) Reduction in VEGF-A expression by PARVA silencing. The VEGF-A expression of the scrambled control (si-NC) and PARVA-silenced A549 transfectants (si-PARVA-2) was detected by Western blotting. β-Actin was used as the loading control. Western blot experiments were repeated at least three times.

Microvessel density (MVD) is a useful measurement to assess the neovasculature in tumours [21]. To investigate whether PARVA promotes tumour growth by upregulating angiogenesis, the MVD of the tumours was measured by immunohistochemistry staining of anti-CD31 in the mice implanted subcutaneously with mock or PARVA-overexpressing cells. The MVD of each sample represents the mean value of three individual IHC slides from microvessel counts of five fields. We observed a higher intensity of brown colour in the PARVA-overexpressing tumours compared with the mock tumours (Fig. 3A, right panel). The quantitative MVD values were higher for the PARVA group compared with the mock group (Fig. 3A, left panel).

To examine further whether PARVA induces angiogenesis directly, we collected the serum-free cultured medium of mock or stably pooled PARVA-overexpressing CL1–0 transfectants and incubated endothelial cells in this medium for 4 hours. The PARVA angiogenesis-promoting activity was measured by quantifying the branch points and total tube lengths with the angiogenesis module of the MetaXpress software. PARVA increased the number of branch points and tube lengths (Fig. 3B). In parallel, the silencing of endogenous PARVA decreased tube-forming activity (Fig. 3C). Western blot analysis of the CL1–0 and A549 cells showed that more vascular endothelial growth factor-A (VEGF-A), one of the most potent angiogenic factors, was produced in PARVA-overexpressing cells compared with the mock control cells (Fig. 3D and E, left panels). A significant amount of secreted VEGF-A was also detected in PARVA-overexpressing cells compared with the mock cells (Fig. 3D, right panel). By contrast, VEGF-A expression was reduced in PARVA-silenced cells compared with the scrambled siRNA control cells (Fig. 3E, right panel). Taken together, these findings suggest that PARVA increases angiogenesis by upregulating VEGF-A expression and that the increased angiogenesis may increase tumorigenesis.

### PARVA promotes invasiveness by phosphorylating ILK and GSK3β

To investigate the mechanism responsible for the promotion of lung cancer progression by PARVA, we performed expression microarrays to identify the genes significantly altered by PARVA (GEO accession number: GSE50657). A total of 371 differentially expressed genes were subjected to pathway analysis using MetaCore software. The 10 most frequent signalling pathways identified by these analyses are listed in S1 Table. At least six of these pathways have been reported to be involved in invasion/migration and cell death. Thus, it is conceivable that the pathways identified correlate well with the PARVA-regulated cancerous phenotypes.

Next, to focus on the significant modulators involved in the differential expression of the 371 PARVA-altered genes, we applied the transcription factor target model of the MetaCore software to identify the 10 top-ranking transcription factors that may be directly or indirectly affected by PARVA (Table 2). Interestingly, we found that ILK was a common regulator of the seven transcription factors including c-Myc, CREB1, ESR1, AP-1, STAT3, HIF1A, and RelA (star marked in Table 2), although the ILK-specific pathway has not been archived within the MetaCore database. Our microarray analysis indicates strongly that the key pathway affected by the expression of PARVA is the ILK signalling pathway. Hence, we characterized further the influence of PARVA on the ILK pathway.

**Table 2:** Transcription factors and target genes in PARVA-induced networks.

Ranking	Network	*P*
1	SP1	1.74E-172
2	HNF4-alpha	1.54E-167
3	c-Myc*	2.21E-145
4	p53	1.98E-106
5	CREB1*	8.90E-97
6	ESR1 (nuclear)*	8.90E-97
7	AP-1*	6.43E-84
8	STAT3*	8.48E-73
9	HIF1A*	2.06E-70
10	RelA (p65 NF-kB subunit)*	1.20E-65

* ILK related-transcription factors and target genes

**Fig 4:**
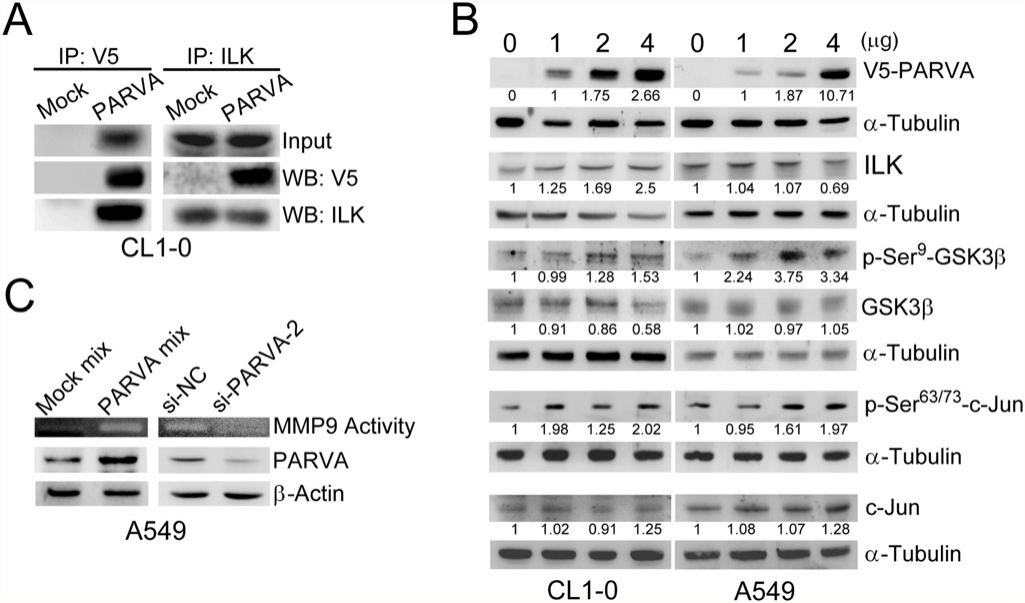
PARVA associates with ILK and induces phosphorylation of GSK3β and MMP9 activity. (A) The interaction between PARVA and ILK. CL1–0 cells were transiently transfected with the V5-PARVA expression vector. V5-PARVA and endogenous ILK were pulled down using anti-V5 and anti-ILK antibodies, respectively, and analysed by Western blotting with the indicated antibodies. (B) The effect of PARVA on GSK3β activity and c-Jun. CL1–0 and A549 cells were transiently transfected with the PARVA-expressing vector. The phosphorylation and protein expression levels were measured by Western blotting with the indicated antibodies. (C) MMP9 activity was positively regulated by PARVA. A549 cells were transiently transfected with the PARVA-expressing vector or siRNA against PARVA (si-PARVA-2). The activity of MMP9 was detected using a zymography assay. The intensity of the blank zone in the gels represents the activity of MMP9. β-Actin was used as the loading control.

PARVA is a component of the PIP complex, which induces several signalling pathways predominantly by phosphorylating Akt and GSK3β. Previous studies have shown that overexpression of ILK results in the phosphorylation of Akt/PKB and GSK3β [25] and that the phosphorylation of GSK3β at Ser9 inhibits the activity of GSK3β followed by activation of the activator protein 1 (AP-1) transcription factor complex [26]. First, we found by immunoprecipitation assays that PARVA interacted with ILK in CL1–0 lung cancer cells (Fig. 4A). To determine whether PARVA can inhibit GSK3β and activate AP-1, we performed Western blot analysis. The overexpression of PARVA increased the phosphorylation of GSK3β and c-Jun in both CL1–0 and A549 cells (Fig. 4B). AP-1 is one of the key transcription factors that can transcriptionally activate MMP9 [27,28,29]. As expected, zymography assays showed that MMP9 activity was increased by the overexpression of PARVA but was inhibited by the knockdown of endogenous PARVA using PARVA-specific siRNA (Fig. 4C).

**Fig 5:**
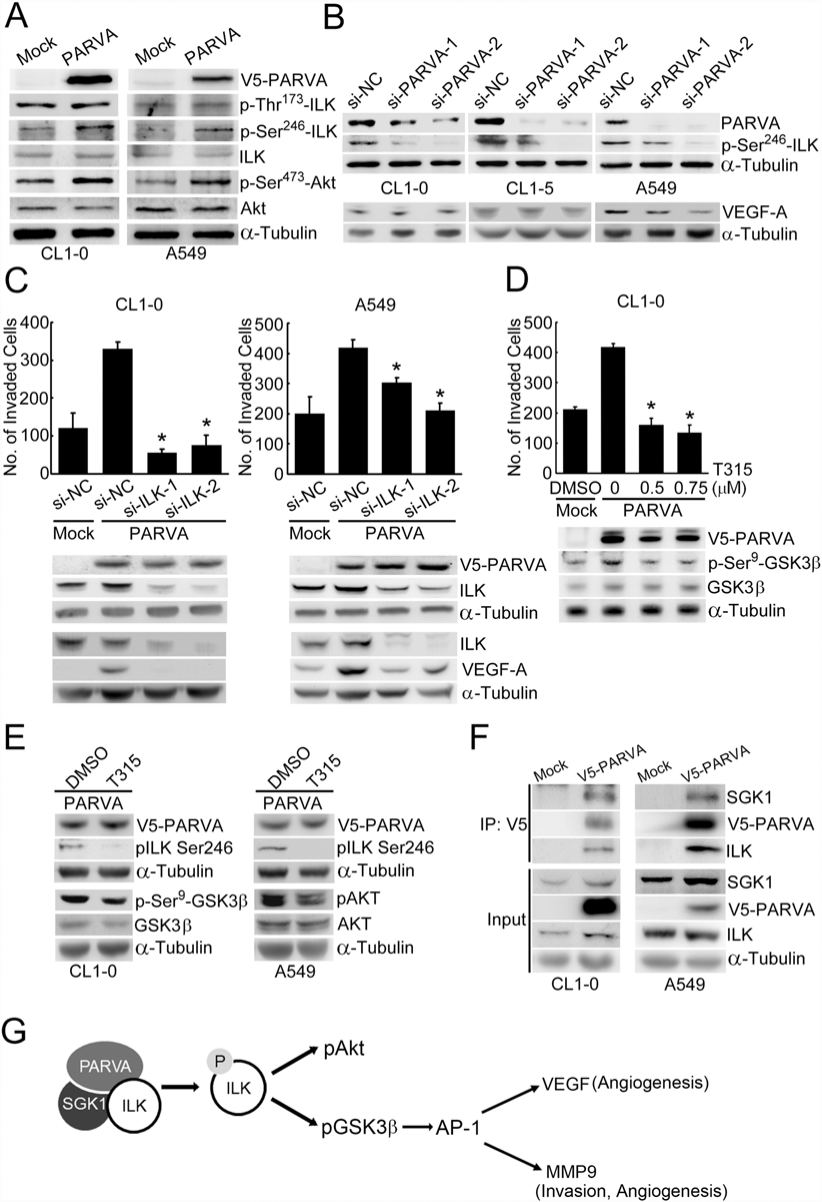
PARVA-induced invasion and VEGF-A expression are mediated by ILK activation. (A) The effect of PARVA on the activities of ILK and Akt. CL1–0 and A549 cells were transiently transfected with the PARVA-expressing vector. The phosphorylation and protein expression levels were measured by Western blotting with the indicated antibodies. ILK phosphorylation was measured using anti-pILK Ser246 and anti-pILK Thr173 antibodies. (B) PARVA silencing attenuates ILK phosphorylation at Ser246 and VEGF-A expression. Cells were transfected with scrambled control (si-NC) and PARVA-specific siRNA (si-PARVA-1 and si-PARVA-2). ILK phosphorylation and VEGF-A expression were detected by Western blotting. α-Tubulin was used as the loading control. (C) Silencing ILK blocks PARVA-induced invasion. The expression of ILK was knocked down by introducing siRNAs against ILK (si-ILK-1 and si-ILK-2) in V5-PARVA-overexpressing cells, and the invasive ability of the cells was analysed by Boyden chamber assays. The expression of ILK and VEGF-A was detected by the indicated antibodies. *, *P* < 0.05, compared with the scrambled control (si-NC). (D) ILK inactivation blocks PARVA-induced invasion. ILK activity was inhibited by an ILK inhibitor, T315. CL1–0 cells were transfected with the PARVA-expressing vector, treated with 0, 0.5 or 0.75 μM T315 and analysed by invasion assay. Cells treated with 0.1% DMSO served as the control vehicle. *, *P* < 0.05, compared with the PARVA-overexpressing cells treated with DMSO. α-Tubulin was used as the loading control. (E) ILK inhibitor blocks PARVA-mediated phosphorylation of ILK and downstream activity. V5-PARVA-transfected CL1–0 and A549 cells were treated with 1 μM of ILK inhibitor T315 for 24 hrs. Cells treated with 0.1% DMSO served as the vehicle control. Cell lysates were subjected to Western blot with the designated antibodies. *, *P* < 0.05, compared to the DMSO control. α-Tubulin was used as the loading control. (F) PARVA can interact with ILK and SGK1. CL1–0 and A549 cells were transiently transfected with the V5-PARVA construct or an empty vector (mock). PARVA was immunoprecipitated using V5 antibody followed by Western blot with the indicated antibodies. Input lanes were loaded with 1% of cell lysate. α-Tubulin was used as a loading control. The immunoprecipitation experiments were repeated three times. (G) Schematic showing the PARVA enhanced phosphorylation of ILK and activation of Akt and GSK3β signalling. The overexpression of PARVA increases ILK phosphorylation at Ser246, and in turn, phosphorylated ILK activates Akt and inhibits GSK3β activity, leading to the upregulation of the downstream genes AP-1 (c-Jun), VEGF and MMP9, and the promotion of angiogenesis and metastasis.

In this study, we first discovered that PARVA can activate the GSK3β—AP-1–MMP9 pathway in lung cancer cells. These results indicated that PARVA might promote invasion and metastasis by increasing ILK activity and by activating the GSK3β—AP-1–MMP9 pathway. We next examined whether ILK activity can be modulated by the expression of PARVA. A previous study indicated that ILK was activated by phosphorylation at Ser246 and Thr173, and that this phosphorylation resulted in significantly increased migration [30]. We used phosphorylation-specific antibodies to examine the phosphorylation of ILK at residues Ser246 and Thr173. The expression level of PARVA did not influence ILK at the protein level but increased its phosphorylation at Ser246 as well as the phosphorylation of a downstream signalling mediator, Akt, in both CL1–0 and A549 cells (Fig. 5A). To verify the regulatory role of PARVA in ILK Ser246 phosphorylation, the endogenous PARVA was silenced by two different PARVA-specific siRNAs in CL1–0, CL1–5 and A549 cells. Knockdown of PARVA markedly inhibited the phosphorylation of ILK at Ser246. In addition, silencing PARVA led to a decrease in VEGF-A expression in A549 cells but not significantly in CL1–0 or CL1–5 cells (Fig. 5B).

To analyse whether PARVA-induced phenotypes are mediated predominantly via the ILK signalling pathway, we examined the contribution of ILK to PARVA-induced invasion. In the presence of PARVA expression, the silencing of ILK expression abrogated the increased invasion induced by PARVA overexpression compared with the expression of scrambled control siRNA in CL1–0 and A549 cells (Fig. 5C, upper panel). To investigate whether PARVA-induced VEGF-A was influenced by ILK, we silenced ILK in the constitutively PARVA-overexpressing cells and measured the expression of VEGF-A. Silencing of ILK led to a decrease in PARVA-induced VEGF-A expression (Fig. 5C, lower panel). In addition, inhibition of the kinase activity of ILK using the ILK inhibitor T315 [31] significantly reduced the invasive ability and the extent of GSK3β phosphorylation in the PARVA-overexpressing transfectants in a dose-dependent manner (Fig. 5D). We also demonstrated that the reduction of ILK phosphorylation at Ser246 was associated with the down-regulated phosphorylation of its substrates, GSK3β and AKT respectively in CL1–0 and A549 cells (Fig. 5E). These results strongly suggested that PARVA modulates invasiveness by activating ILK signalling. Next, to clarify whether PAK1, a kinase that can phosphorylate ILK, is involved in PARVA-mediated ILK phosphorylation, we silenced endogenous PAK1 by lentivirus-based shRNA approach in constitutively PARVA-overexpressing cells. The results showed that the decrease of endogenous PAK1 inhibited ILK phosphorylation at S246 (S3 Fig.), suggesting that PAK1 may at least regulate the PARVA-induced phosphorylation of ILK. Furthermore, we determined the PARVA-induced protein-protein interaction between ILK and PAK1 by using endogenous and exogenous immunoprecipitation assay. The results revealed that PAK1 might not interact with ILK even under the induction of PARVA (S4 Fig.). We further inspected the other potential kinase, serum and glucocorticoid induced kinase 1 (SGK1) which had been demonstrated to involve in phosphorylating ILK at Thr181 and Ser246 [32]. The results of immunoprecipitation assay indicated that endogenous SGK1 could associate with endogenous ILK and exogenous V5-PARVA proteins in CL1–0 and A549 cells (Fig. 5F).

## Discussion

A previous report has shown that PARVA is highly expressed in most chondrosarcomas, whereas PARVA is almost undetectable in normal cartilage, indicating that PARVA might be involved in malignant transformation [6]. Although PARVA has been shown to participate in the regulation of the actin cytoskeleton and survival of human cells, limited studies have explored the role of PARVA in cancer progression and the underlying mechanism. In this study, we found that PARVA increased the invasion, colony formation and tube formation of lung cancer cells *in vitro*, and tumorigenesis and metastasis *in vivo*. These findings suggest that PARVA can act as an oncogene during lung cancer progression.

Our data indicate that PARVA increases *in vitro* clonogenesis and *in vivo* tumorigenicity but does not affect *in vitro* cell proliferation. Similar results have also been observed for α-catulin, another ILK-interacting protein, which induced tumour growth *in vivo* but had no effect on cell proliferation *in vitro* [8], suggesting that increased colony numbers occur because of a change in the colony-forming ability but not cell proliferation. By contrast, tumorigenicity relies on the nature of cancer cells and on contributions from the tumour microenvironment, including the surrounding blood vessels, immune cells, fibroblasts, ECM, signalling molecules and other cells [33,34]. Therefore, tumour-associated endothelial cells may play a pivotal role in the tumour microenvironment and cancer progression. Several angiogenic factors, such as VEGF-A and MMPs, have been shown to induce endothelial cell growth and to increase vascular permeability. In vascular smooth muscle cells (vSMCs), PARVA plays a crucial role in vascular development and stability by downregulating Rho/ROCK signalling [35]. The absence of PARVA–ILK complexes has been shown to reduce the level of MLC2 phosphorylation, leading to a loss of vSMC contractile ability [36].

Previous studies have indicated that PARVA may induce angiogenesis by regulating ILK [37]. In this study, the ectopic expression of PARVA activated and inhibited Akt and GSK3β (the downstream mediators of ILK), respectively (Fig. 4B and 5A). Moreover, PARVA induced VEGF expression, promoted tube formation and increased the expression and phosphorylation of c-Jun (Fig. 3D and 4B). It is well known that VEGF can be induced via the AP-1 pathway in different situations [37,38]. Our data suggest that PARVA-overexpressing cancer cells promote tumour angiogenesis by stimulating the ILK–Akt–VEGF signalling pathway (Fig. 3E, 5B and 5C). Similar signalling was also reported in prostate cancers in which ILK increases angiogenesis via the Akt–HIF-1–VEGF pathway [39]. However, our data showed that the downregulation of VEGF-A by silencing of PARVA was remarkably observed in A549 cells but not in CL1–0 or CL1–5 cells. Because these three cell lines all represent an adenocarcinoma cell type, we speculate that their genotypes differ. For instance, CL1–0 and CL1–5 contain the p53 mutation genotype but A549 cells have wild-type p53 [22]. Moreover, CL1–5 and CL1–0 have an exon 5 deletion mutation on the *PTEN* gene, whereas A549 cells have the wild-type *PTEN* genotype [40]. These different genotypes may influence the signalling pathway of PARVA-mediated VEGF expression. Further investigation is needed to confirm this hypothesis.

To investigate further the mechanism underlying the PARVA-mediated cancer phenotypes, we applied expression microarray analysis to identify the differentially expressed genes in PARVA-overexpressing cells compared with mock cells. Of the top-ranking pathways altered by PARVA (S1 Table), we focused on a muscle contraction-related pathway. Previous reports have shown that the aVbeta3–PI3K–Akt–eNOS–NO signalling pathway may be involved in promoting cancer invasion and angiogenesis [41,42]. In addition, the oncostatin M-, leptin- and growth hormone-triggered pathways share the common downstream PI3K–Akt and MAPK cascades. Leptin has also been shown to increase angiogenesis by increasing the activation of NADPH oxidase and the expression of MMP9 in Sca-1^+^/Flk-1^+^ vascular progenitor cells [43]. The angiogenic and invasive effects of PARVA-induced signalling in cancer cells might be attributed to the PI3K–Akt pathway rather than to the increase in NADPH expression, although the MMP9 level was elevated in these cells (Fig. 4A and 5A).

In general, cancer-associated pathways are altered by inducing intracellular signalling mediators such as ERK, PI3K, Akt and GSK3β. Interestingly, we found that ILK was a common regulator of these seven transcription factors (Table 2), and we found that ILK was the most potent mediator in this study. Previous studies have shown that PIP induces the phosphorylation of Akt/PKB and GSK3β, and the enforced expression of ILK induces the phosphorylation of Akt and GSK3β [11,44]. Because PARVA overexpression can affect Akt and GSK3β, PARVA might also activate ILK or contribute to the formation of PIP complexes, followed by phosphorylation of Akt and GSK3β. PARVA bound to active ILK, whose activity was detected by measuring phosphorylated GSK3β Ser9 but not phosphorylated ILK [11]. Our results show, for the first time, that PARVA significantly alters the phosphorylation of ILK, particularly at Ser246 (Fig. 5A and B), which has been reported as an important activation site of ILK [30]. In addition, the decrease of ILK phosphorylation reduced the activity of downstream substrates, suggesting ILK phosphorylation might link to its kinase activity and influence the regulation of downstream signalling (Fig. 5E). Previous studies indicated that the phosphorylation status affects ILK kinase activity, which correlates significantly with its biological functions such as nuclear localization, cellular invasion and angiogenesis [30,45]. The activity of ILK kinase was found to be significantly decreased in PARVA-depleted prostate cancer cells [11], suggesting that the formation of PARVA–ILK complexes is required for the activation of ILK. Thus, PARVA might contribute to cancer progression by activating ILK.

The interaction between ILK and PARVA, and the ILK-mediated Akt phosphorylation of Ser473, were abolished by mutating ILK [11,44]. We characterized the significance of ILK in PARVA-regulated invasion. Both the knockdown of ILK expression and inhibition of ILK activity markedly decreased the PARVA-induced invasive capacity of the cancer cells (Fig. 5C and D). Next, we focused on the influence of PARVA on the activity of ILK. Previous reports revealed two kinases that can potentially phosphorylate ILK. One is p21-activated kinase 1 (PAK1), which can phosphorylate ILK at Thr173 and Ser246 [30,32]. PAK1 is involved in the regulation of PARVA-dependent matrix degradation by facilitating the formation of complexes containing Rac1/Cdc42 GEF beta-PIX and PARVA [18]. These studies suggested that PARVA recruits PAK1 to phosphorylate ILK, thereby activating downstream signalling. Nevertheless, our data showed that PAK1 might not interact with ILK in lung cancer cells even though silencing PAK1 decreased the PARVA-induced phosphorylation of ILK at Ser246 (S3 and S4 Fig.). The other kinase is SGK1, which can phosphorylate ILK at Thr181 and Ser246. Phosphorylation of ILK at Ser246 makes ILK bind to 14–3–3, resulting in blocking ILK kinase activity [32]. Here, our data suggest that PARVA, ILK, and SGK1 can form a protein complex, leading to ILK phosphorylation. Nevertheless, the relationship between PARVA and SGK1 remains unknown. Further investigations are needed to clarify it.

In conclusion, our results demonstrate clearly, for the first time, that PARVA increases metastasis, tumorigenicity and angiogenesis of lung cancers. These effects of PARVA have not been characterized previously. We provide evidence that the PARVA-mediated regulation of these functions in lung cancers occurs through the activation of ILK, although we cannot exclude other pathways that may also contribute to these effects. Our findings suggest that PARVA may be a promising target for the development of targeted therapy in lung adenocarcinoma.

## Supporting Information

S1 Table Top 10 potentially PARVA-regulated pathways. (DOC)

S1 Fig PARVA promotes migration in lung cancer cells. (DOC)

S2 Fig PARVA does not influence cell proliferation in lung cancer cells. (DOC)

S3 Fig PAK1 silencing decreases ILK phosphorylation at Ser246. (DOC)

S4 Fig PAK1 may not form protein complex with PARVA and ILK. (DOC)
