# Supplementary material for: PARVA Promotes Metastasis by Modulating ILK Signalling Pathway in Lung Adenocarcinoma
Source: PLoS One. 2015 Mar 4;10(3):e0118530. doi: 10.1371/journal.pone.0118530 (PMC4349696; doi:10.1371/journal.pone.0118530)
Supplement: S1 Table — (DOC) [file pone.0118530.s001.doc]

**Table S1.** Top 10 potentially PARVA-regulated pathways.

| **Ranking** | **Pathway** | ***P*** |
| --- | --- | --- |
| 1 | Muscle contraction: Regulation of eNOS activity in endothelial cells* | 6.39E-07 |
| 2 | IL-2 activation and signaling pathway^#^ | 3.70E-05 |
| 3 | Oncostatin M signaling via MAPK* | 1.87E-04 |
| 4 | Growth hormone signaling via PI3K/AKT and MAPK cascades* | 3.08E-04 |
| 5 | Corticoliberin signaling via CRHR1 | 6.04E-04 |
| 6 | Apoptosis and survival: NO synthesis and signaling^#^ | 8.68E-04 |
| 7 | Leptin signaling via JAK/STAT and MAPK cascades* | 9.34E-04 |
| 8 | IL-3 activation and signaling pathway^#^ | 1.77E-03 |
| 9 | Apoptosis and survival: Role of CDK5 in neuronal death and survival^#^ | 2.31E-03 |
| 10 | Reproduction: GnRH signaling | 2.34E-03 |

*Invasion and angiogenesis-related pathways

^#^Cell death-related pathways
