# Supplementary material for: PARVA Promotes Metastasis by Modulating ILK Signalling Pathway in Lung Adenocarcinoma
Source: PLoS One. 2015 Mar 4;10(3):e0118530. doi: 10.1371/journal.pone.0118530 (PMC4349696; doi:10.1371/journal.pone.0118530)
Supplement: S1 Fig — (DOC) [file pone.0118530.s002.doc]

**S1 Fig.**

**
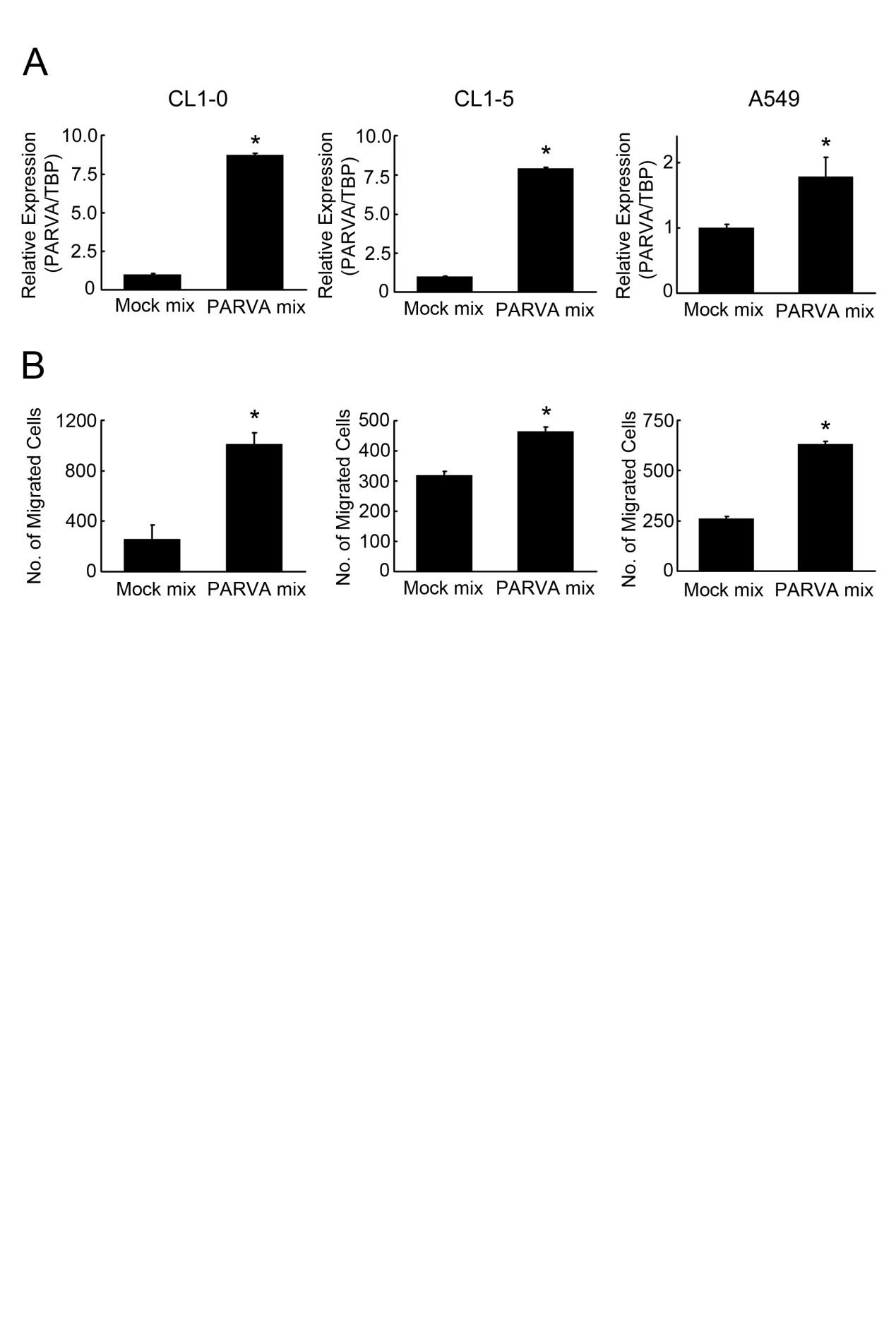
**

**S1 Fig.** PARVA promotes migration in lung cancer cells. (A) PARVA expression levels in the pooled, stably PARVA-overexpressing lung cancer cells. CL1-0, CL1-5 and A549 cells were stably transfected with the pcDNA3.1/V5-His TOPO-tagged PARVA construct (PARVA mix) or an empty vector (mock mix), and the expression levels of PARVA were determined by real-time RT-PCR. (B) *In vitro* migration abilities of pooled, stably PARVA-overexpressing cells. The migration abilities of stably pooled PARVA-overexpressing CL1-0, CL1-5 and A549 cells were assessed by Boyden chamber assays. ***,** *P* < 0.05, compared to the mock control.
