# Supplementary material for: PARVA Promotes Metastasis by Modulating ILK Signalling Pathway in Lung Adenocarcinoma
Source: PLoS One. 2015 Mar 4;10(3):e0118530. doi: 10.1371/journal.pone.0118530 (PMC4349696; doi:10.1371/journal.pone.0118530)
Supplement: S2 Fig — (DOC) [file pone.0118530.s003.doc]

**S2 Fig.**


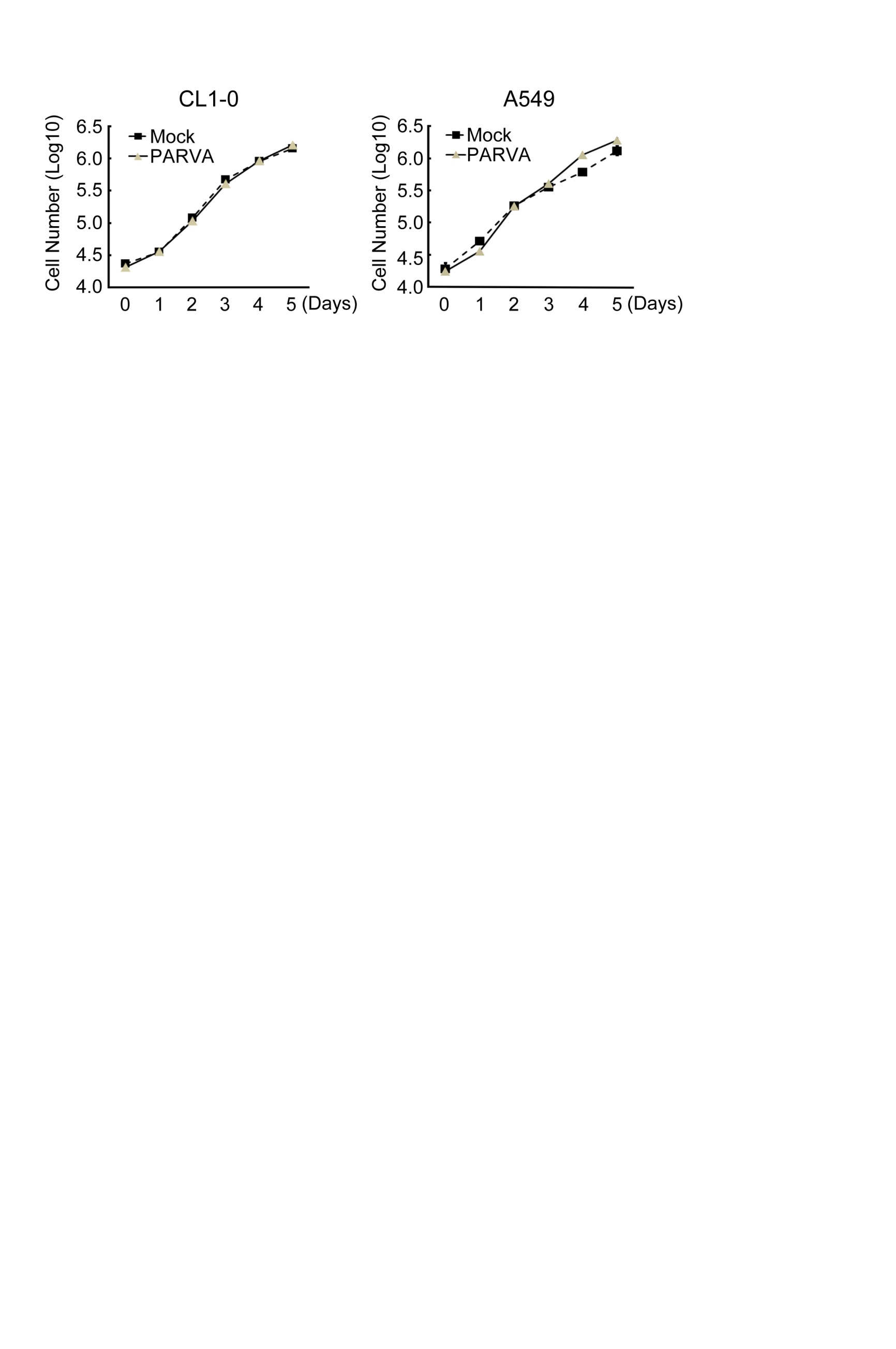


**S2 Fig.** PARVA does not influence cell proliferation in lung cancer cells. Cell proliferation of the pooled, stably PARVA-overexpressing CL1-0 and A549 cells was assessed by cell counting every 24 h for five days. Mock mix and PARVA mix cells were seeded at a density of 1X10^3^ cells per well in 6-well plates.
