# Supplementary material for: PARVA Promotes Metastasis by Modulating ILK Signalling Pathway in Lung Adenocarcinoma
Source: PLoS One. 2015 Mar 4;10(3):e0118530. doi: 10.1371/journal.pone.0118530 (PMC4349696; doi:10.1371/journal.pone.0118530)
Supplement: S3 Fig — (DOC) [file pone.0118530.s004.doc]

**S3 Fig.**

**
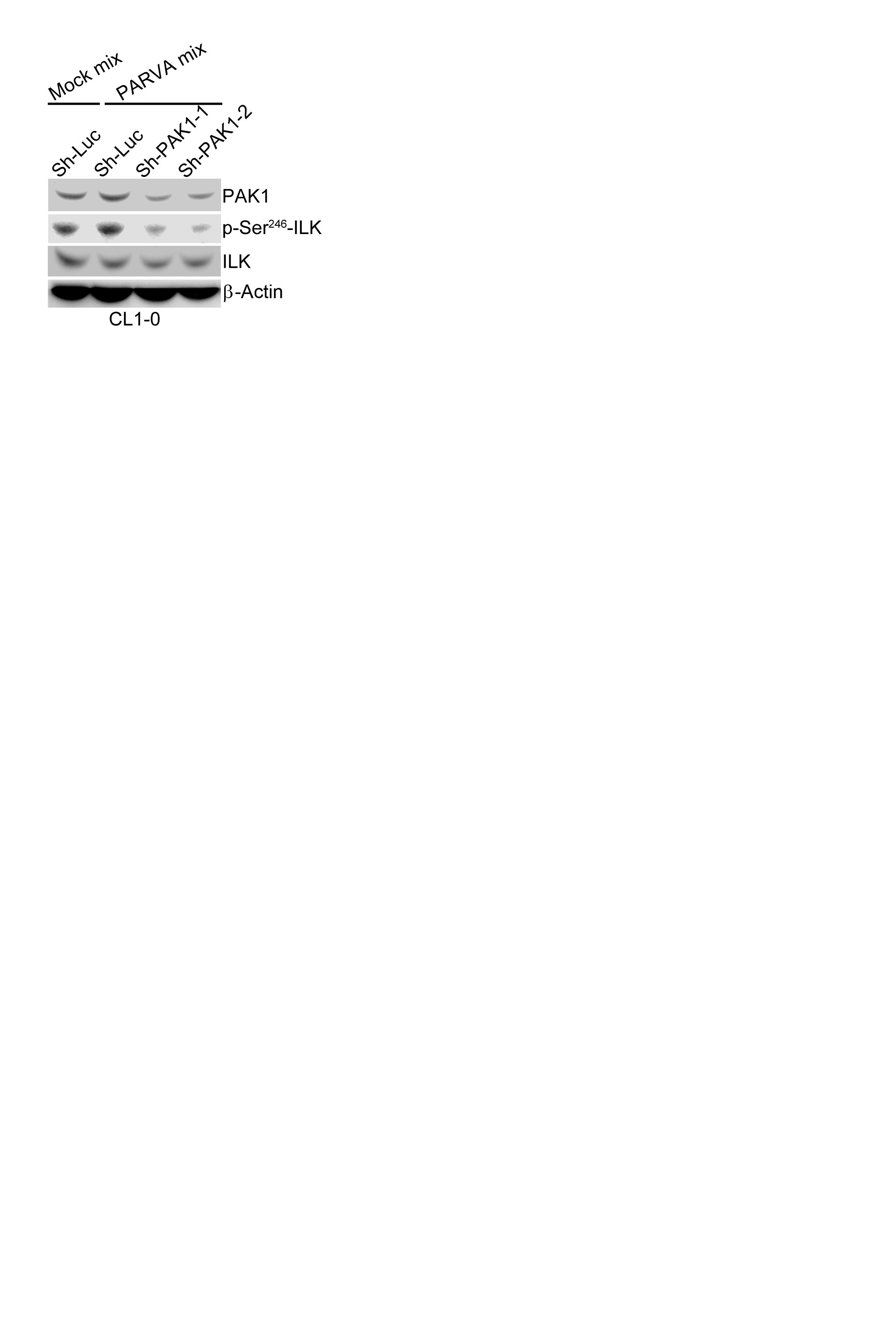
**

**S3 Fig.** PAK1 silencing decreases ILK phosphorylation at Ser246. Endogenous PAK1 was silenced by lentivirus-based shRNA infection in constitutively PARVA-overexpressing cells. The expression levels of proteins were measured by Western blot with the indicated antibodies. β-Actin was used as the loading control.
