# Supplementary material for: PARVA Promotes Metastasis by Modulating ILK Signalling Pathway in Lung Adenocarcinoma
Source: PLoS One. 2015 Mar 4;10(3):e0118530. doi: 10.1371/journal.pone.0118530 (PMC4349696; doi:10.1371/journal.pone.0118530)
Supplement: S4 Fig — (DOC) [file pone.0118530.s005.doc]

**S4 Fig.**


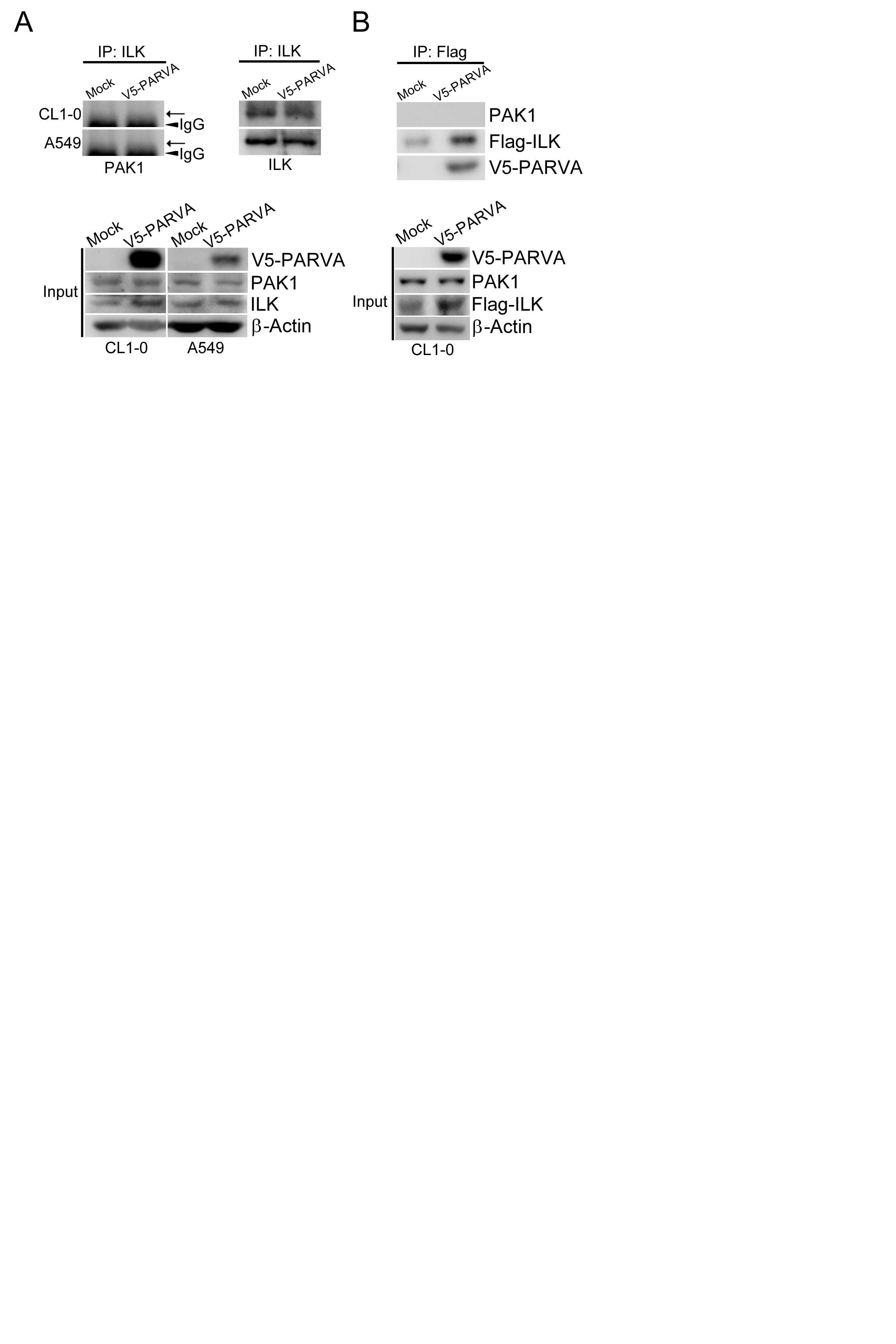


**S4 Fig.** PAK1 may not form protein complex with PARVA and ILK. (A) CL1-0 and A549 cells were transiently transfected with the pcDNA3.1/V5-His TOPO-tagged PARVA construct (V5-PARVA) or an empty vector (mock), and protein-protein interactions were evaluated by Immunoprecipitation assay. Endogenous ILK was pulled down by ILK antibody. The arrow indicated the position of PAK1 and arrow head indicated IgG. (B) Flag-ILK construct was co-transfected with V5-PARVA or mock into CL1-0 cells. Flag-ILK was pulled down by anti-Flag antibody. Input lanes were loaded with 1% of cell lysate. The protein expression levels were measured by Western blot with the indicated antibodies. β-Actin was used as a loading control.
